# The role and mechanism of neutrophils in oncolytic virus therapy

**DOI:** 10.3389/ebm.2026.11192

**Published:** 2026-08-06

**Authors:** Baixian Huang, Qixin Yang, Ruixue Wang, Chaochao Zhao

**Affiliations:** School of Medicine, Foshan University, Foshan, Guangdong, China

**Keywords:** immune response, N1/N2 phenotypes, neutrophils, oncolytic viruses, tumor microenvironment

## Abstract

Oncolytic viruses (OVs) are a class of viruses capable of specifically infecting and killing tumor cells. They exert antitumor effects through direct lysis of tumor cells, activation of the immune system, and disruption of tumor vasculature. Neutrophils, which originate from the bone marrow, are the most abundant leukocytes in peripheral blood and the immune system and play a dual regulatory role in oncolytic virotherapy. In different TME, neutrophils can polarize into two phenotypes: N1 and N2. N1 neutrophils, owing to their chemotaxis and tumor-homing capabilities, can serve as delivery vehicles for OVs, enhance the therapeutic efficacy of oncolytic virotherapy, and activate host immune responses. In contrast, N2 neutrophils impair the efficacy of OVs through their antiviral activity and immunosuppressive functions. Therefore, an in-depth understanding of the mechanisms underlying the role of neutrophils in oncolytic virotherapy is of great significance for optimizing OV-based therapies and improving their clinical application outcomes.

## Impact statement

Neutrophils exert a dual regulatory role in oncolytic virotherapy but remain poorly understood, limiting clinical translation. This review systematically clarifies N1/N2 phenotypic polarization, pro-tumor/anti-tumor mechanisms, and interactions with the TME. It integrates key molecular pathways and emerging intervention strategies to guide rational optimization of OV regimens. This work advances targeted immunovirotherapy, supports precise neutrophil modulation, and promotes safer, more effective clinical application of OV therapy.

## Introduction

The earliest reports of oncolytic viruses (OVs) date back to 1904, when scientists observed an unexpected remission of leukemia symptoms in a female chronic leukemia patient following infection with the influenza virus [1]. Subsequently, in 1912, Italian physician Depace reported spontaneous regression and shrinkage of cervical cancer in a patient after administration of an attenuated rabies vaccine [2]. These observations spurred interest in the potential oncolytic properties of viruses. The evolution of OV research can be broadly divided into three stages. The first stage primarily involved the direct use of wild-type viruses. As early as a century ago, physicians attempted to inject live viruses into cancer patients, aiming to combat tumors through a “poison against poison” strategy. Although these early efforts yielded transient oncolytic effects, they often resulted in significant toxicities due to wild-type virus infection [3]. In the 1990s, advances in gene editing enabled targeted modification of wild-type viral genomes, allowing for tumor-specific replication. While most of these modified OVs exhibited limited efficacy, their toxicity profiles were markedly reduced, thereby establishing a safety foundation for subsequent development. The third stage, initiated in the current century, is characterized by the insertion of exogenous genes into OV to enhance their therapeutic effects. In 2015, Talimogene laherparepvec became the first OV therapy to receive regulatory approval worldwide [4], and since then, an increasing number of OV candidates have entered clinical trials.

### Overview of oncolytic viruses

**FIGURE 1:**
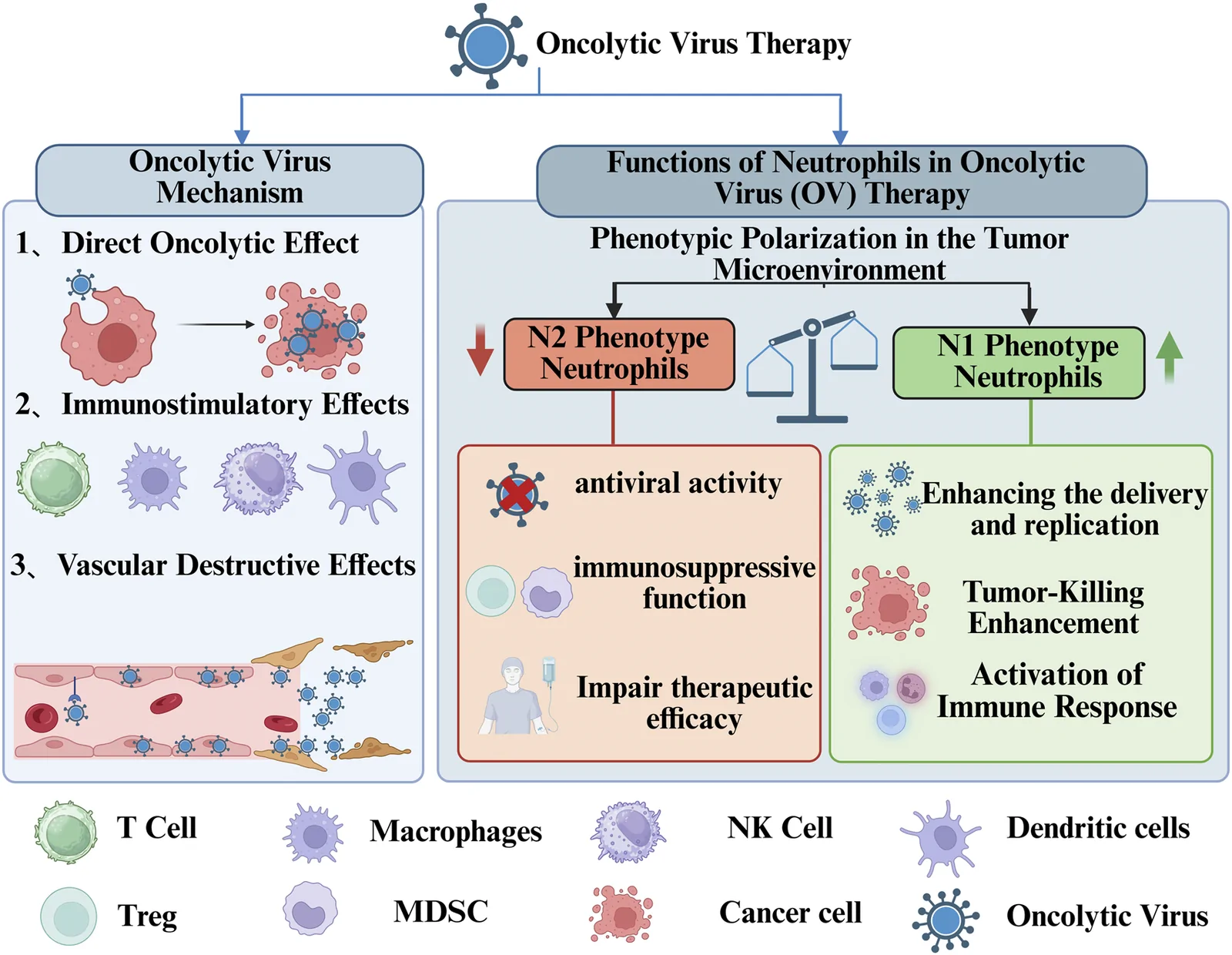
Oncolytic virus therapy and neutrophil. Created with BioGDP.com [8].

OVs are defined as viruses capable of specifically infecting and replicating within tumor cells, ultimately inducing tumor cell lysis and death. The fundamental principle of this therapeutic approach lies in the selective infection of tumor cells by OVs. Beyond their direct cytolytic effect on tumor cells [5], these viruses can further potentiate antitumor immunity by activating the host immune system [6]. Compared with conventional treatment modalities, OVs therapy offers precise targeting of tumor tissues while causing minimal damage to adjacent healthy tissues. Additionally, OVs can remodel the tumor immune microenvironment and enhance the host’s immune response [7] as shown as Figure 1. Collectively, these properties position OV therapy as a promising strategy capable of achieving more durable and broad-spectrum efficacy in cancer treatment. Currently, a variety of OVs have entered clinical trials, as summarized in Supplementary Table 1.

OVs can be categorized based on their nucleic acid composition into double-stranded DNA viruses, double-stranded RNA viruses, positive-sense single-stranded RNA viruses, and negative-sense single-stranded RNA viruses [9]. According to their origin, they are primarily divided into two classes: naturally occurring viruses and genetically engineered viruses [10]. Although naturally occurring viruses are capable of selectively infecting and killing tumor cells, they are often limited by low therapeutic efficacy and insufficient safety. In contrast, genetically engineered viruses, through the insertion or deletion of specific genes, exhibit enhanced selectivity and replication capacity within tumor cells while reducing damage to normal tissues. Moreover, these modifications enable OVs to evade recognition and clearance by the host immune system, thereby prolonging their persistence in the body and improving therapeutic outcomes [11].

### Overview of neutrophils

Neutrophils (also known as neutrophil granulocytes or neutrophilic leukocytes) originate from the bone marrow [12] and represent the most abundant type of white blood cells in peripheral blood and the immune system, accounting for approximately 50%–70% of total leukocytes in mammalian blood [13]. With the development of the tumor microenvironment (TME) concept, studies have revealed the infiltration of various immune cell types within the TME, among which T lymphocytes and macrophages have been extensively investigated. In recent years, neutrophils have also garnered considerable attention. Research indicates that neutrophils can polarize into distinct phenotypes depending on the TME context. Neutrophil phenotype 1 (N1) typically appears during the early stages of tumor development and primarily functions to inhibit tumor growth. In contrast, neutrophil phenotype 2 (N2) is more prevalent in later stages and predominantly promotes tumor progression [14] (Figure 1). Therefore, inhibiting the polarization of neutrophils toward the N2 phenotype while promoting their conversion to the N1 phenotype represents a key strategy in tumor therapy. For instance, Yasufumi Kaneda and colleagues demonstrated that inactivated Sendai virus envelopes can polarize neutrophils toward the N1 phenotype. N1 neutrophils exhibit antitumor properties and enhance cytotoxic T lymphocyte activity, thereby suppressing tumor growth [15]. Under specific conditions, N1 and N2 phenotypes can interconvert. For example, transforming growth factor-beta (TGF-β) regulates the transition of neutrophils to the N2 phenotype [14]. In a pancreatic cancer liver metastasis model, TGF-β secreted by tumors activates SMAD3 signaling pathway in neutrophils, induces the expression of nuclear factor erythroid 2, and subsequently activates peptidyl-arginine deiminase 4, driving neutrophil polarization toward the N2 phenotype and promoting tumor metastasis [16]. Furthermore, neutrophils can interact with cancer cells to facilitate tumor growth. In lung cancer, tumor cells secrete factors that increase the number of osteocalcin-positive osteoblasts, which in turn activate SiglecF-expressing neutrophils, thereby promoting lung cancer progression [17]. Beyond the N1/N2 polarization model mentioned above, a growing number of studies have revealed the high heterogeneity of neutrophils within the TME. Multiple neutrophil subsets with distinct functions have now been identified, including low-density neutrophils [18], antigen-presenting neutrophils [19], aged neutrophils [20], single-cell transcriptome-defined subsets [21], and they play very different, sometimes even opposite roles in cancer therapy. A detailed summary is provided in Supplementary Table 2.

## Oncolytic virus mechanism

### Direct oncolytic effect

Genetically engineered OVs are capable of specifically targeting tumor cells and selectively replicating within them, ultimately inducing tumor cell lysis. For instance, Teserpaturev (G47Δ), an OV approved for clinical use in Japan, exerts its direct oncolytic effect by targeting aberrant signaling pathways in gliomas [22]. In the early stages of glioma cell infection, herpes simplex virus type 1 (HSV-1) enhances the antitumor efficacy of oncolytic HSV-1 (oHSV-1) in glioma-bearing xenograft mouse models by selectively reducing the expression of the METTL14 protein [23].

### Immunostimulatory effects

As pathogen-associated molecular patterns, OVs can activate innate immune cells, including macrophages and natural killer cells, thereby stimulating a non-specific immune response in the host [24]. OVs engineered to express immunomodulatory genes—such as interleukin-21 and granulocyte-macrophage colony-stimulating factor (GM-CSF)—can promote T cell activation and subsequently trigger an adaptive immune response. For example, Talimogene laherparepvec, which carries the GM-CSF gene, enhances T cell-mediated immunity by recruiting and activating dendritic cells, thereby inducing antitumor responses both at the injection site and systemically [25]. Studies have shown that oncolytic herpes simplex viruses potentiate antitumor immunity by increasing the expression of interferon-stimulated genes, promoting the accumulation of endogenous Z-RNA, and activating Z-DNA binding protein 1, which in turn triggers Z-DNA binding protein 1-mediated PANoptosis, a distinct form of innate immune inflammatory cell death [26]. Newcastle disease virus (NDV) has been developed as an effective OV agent, capable of selective replication in tumor cells, inducing costimulatory activity in T cells, activating macrophages, and exhibiting multiple immunostimulatory functions [27]. In a mouse model of transplanted digestive system tumors, esophageal and colorectal cancer cells demonstrated a robust cytotoxic response to the attenuated NDV strain AMHA1, and this response was positively correlated with the multiplicity of infection [28]. Additionally, OV-mOX40L—constructed by inserting mouse OX40L into HSV-1—has been shown to increase the proportion of N1 neutrophils while decreasing that of N2 neutrophils, thereby inhibiting tumor activity in the treatment of pancreatic ductal adenocarcinoma [29].

### Vascular destructive effects

OV can directly infect and lyse tumor vascular endothelial cells by recognizing specific surface receptors, such as CD46 and CD155, thereby inducing the collapse of vascular structures. For example, the EV-A71-miR124T nanocapsules developed by the team at the Wuhan Institute of Virology are capable of crossing the blood–brain barrier and selectively releasing OVs at the tumor site. This strategy enables precise delivery of OVs to the vascular endothelial cells of gliomas, disrupting tumor blood vessels while preserving the integrity of vessels in normal brain tissue, thus achieving targeted killing of glioma cells [30]. Additionally, the NDV-GT OV developed by the team at Guangxi Medical University induces the expression of the porcine α1,3GT gene on the surface of tumor cells, leading to the generation of α-Gal antigens. In clinical trials, this mechanism results in tumor vascular embolism and necrosis, with a disease control rate of 90%. Concurrently, NDV-GT activates the immune system, inducing substantial infiltration of macrophages and neutrophils around tumor blood vessels, which subsequently secrete pro-inflammatory factors such as tumor necrosis factor-alpha (TNF-α), interleukin-1β, and matrix metalloproteinases, further damaging the vascular basement membrane [31]. Moreover, certain OVs can infect tumor vascular endothelial cells and inhibit the expression of angiogenic factors, including vascular endothelial growth factor [32] and fibroblast growth factor, thereby blocking new blood vessel formation.

## The core regulatory role of neutrophils

### Positive regulation mechanism

#### Enhancing the delivery and replication of oncolytic viruses *in vivo*

**FIGURE 2:**
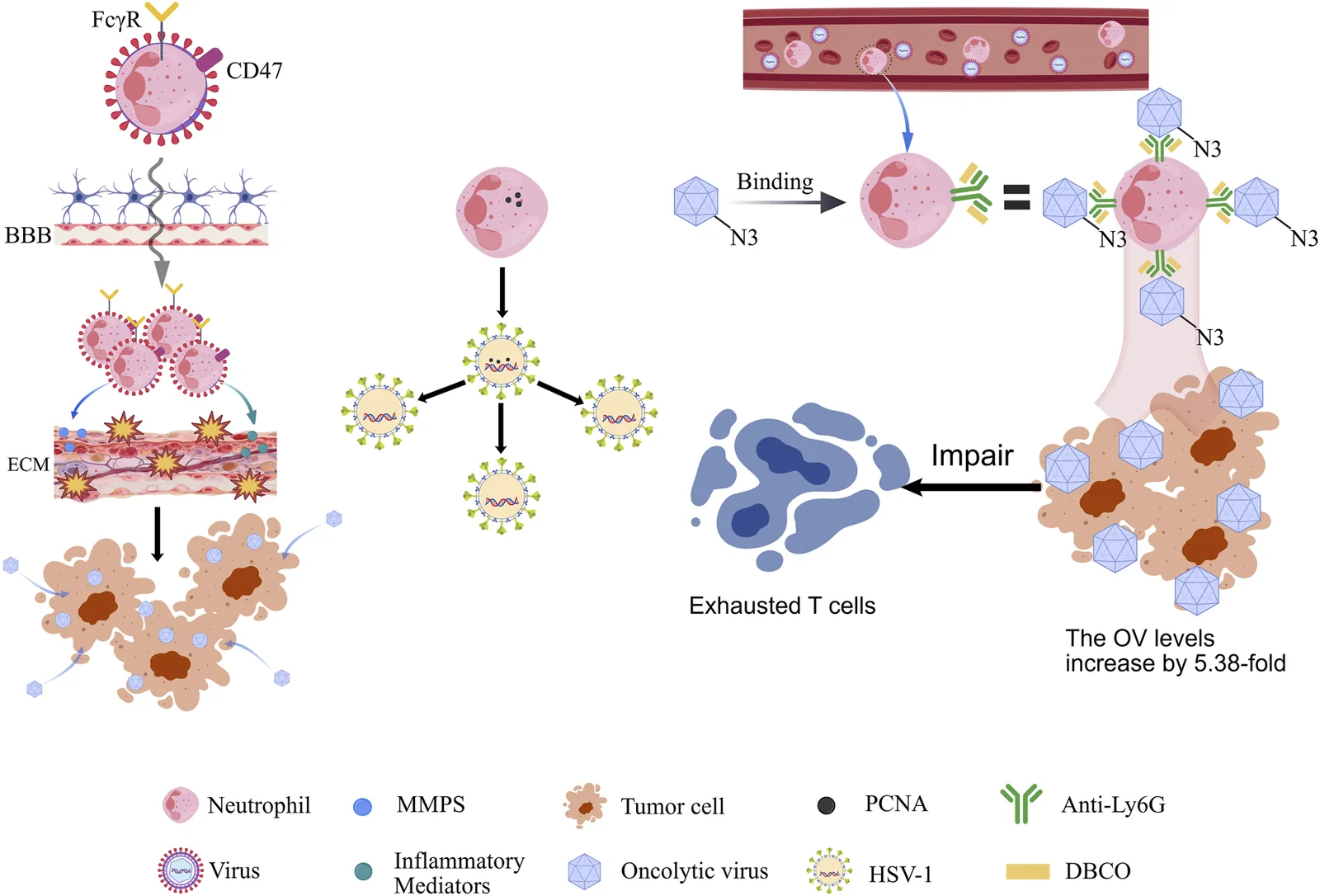
Enhancing the delivery and *in vivo* replication of oncolytic viruses. Created with BioGDP.com [8].

Neutrophils, with their inherent chemotactic and tumor-homing abilities [33], can carry OVs across physiological barriers (such as the blood-brain barrier) or immunosuppressive microenvironments, achieving targeted delivery. After intravenous injection, OVs can be captured by receptors on the surface of neutrophils (such as FcγR), forming a “virus-neutrophil complex”. For example, researchers observed in a clinical trial (ChiCTR2300069323) that increased intratumoral neutrophil infiltration in gastric cancer patients with OV therapy was associated with exacerbated CD8^+^ T cell exhaustion, suggesting that neutrophils may compromise viral efficacy. Inspired by this observation, the investigators designed a bifunctional bioorthogonal delivery system, by using an anti-Ly6G antibody modified with DBCO to tag circulating neutrophils. This approach significantly boosted the virus load inside the tumor. Once the virus reached the tumor site, the neutrophils underwent apoptosis, which relieved immunosuppression and reduced T cell exhaustion, turning neutrophils from a therapeutic obstacle into an effective delivery vehicle [34]. After genetic engineering modification (such as insertion of the CD47 domain), OVs can inhibit the phagocytosis of neutrophils [35], prolong virus survival time, and ensure their effective release at the tumor site. In addition, neutrophils can also destroy tumor vascular basement membranes and extracellular matrix by secreting matrix metalloproteinases and inflammatory factors, further enhancing the penetration ability of OVs into tumors. Recent studies have also found the presence of proliferating cell nuclear antigen in the cytoplasm of neutrophils [36], which plays a key role in the replication process of HSV-1 [37]. In conclusion, Figure 2 illustrates the mechanisms of enhancing the delivery and in vivo replication of oncolytic viruses.

#### Tumor-killing enhancement

In a mouse model of lung melanoma metastasis, treatment with orf virus (ORFV) significantly increased the number of white blood cells, predominantly neutrophils, in the peripheral blood, peritoneal cavity, spleen, liver, and TME. These systemic neutrophils were capable of migrating into the TME and exhibited predominantly an immature phenotype (CD101^-^), with high expression of the chemokine receptor CXCR2 and the activation marker CD69. ORFV-activated neutrophils directly killed tumor cells through the secretion of TNF-α. *In vitro* experiments demonstrated that ORFV-treated neutrophils displayed markedly enhanced cytotoxicity against B16F10 melanoma cells. Moreover, in addition to retaining their intrinsic antigen-presenting capacity, these neutrophils expressed major histocompatibility complex class II(MHC-II), thereby contributing to immunomodulatory functions within the TME [38].

**FIGURE 3:**
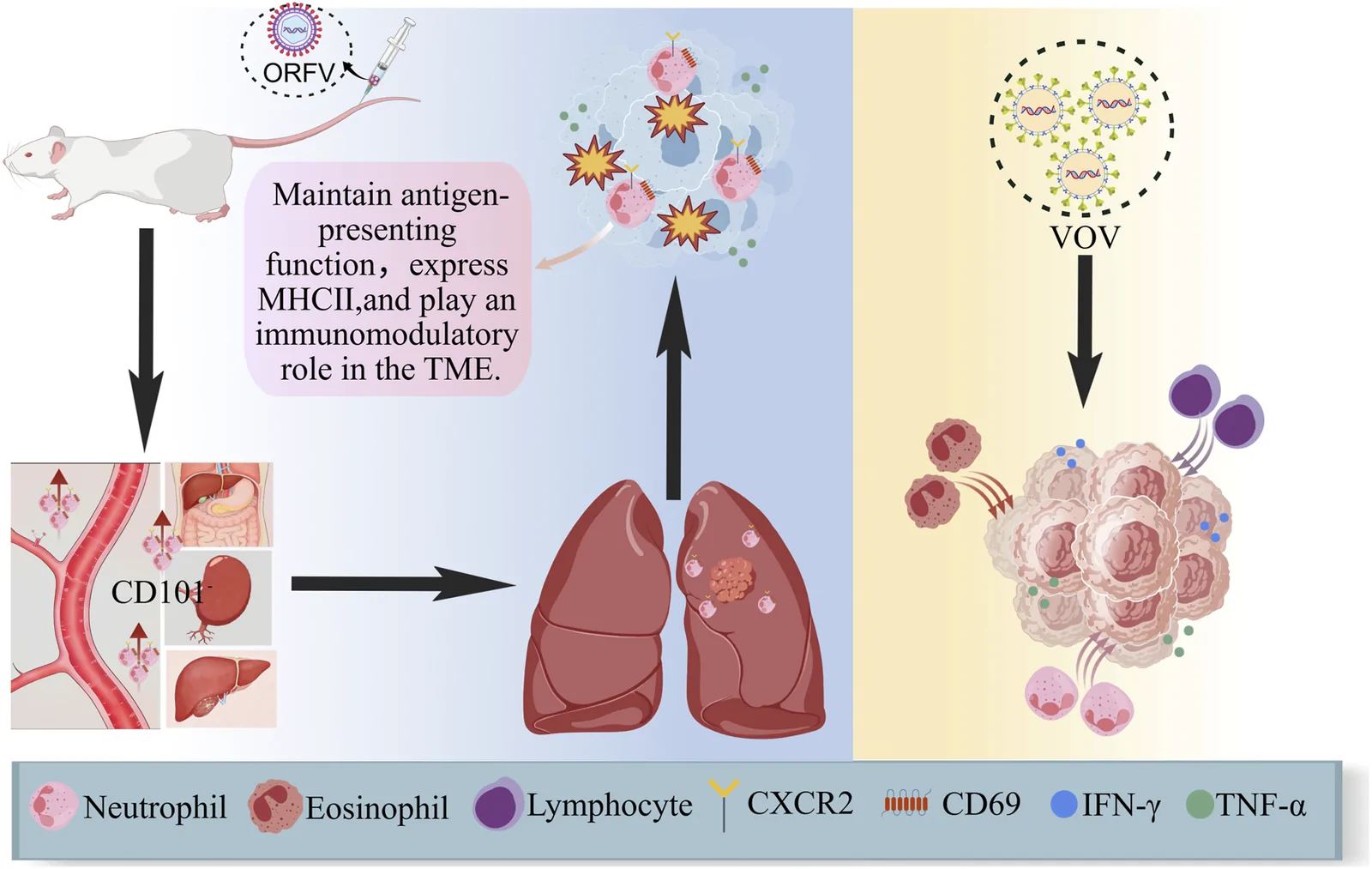
Mechanisms of neutrophil-enhanced tumor killing. Created with BioGDP.com [8].

Similarly, vaccinia virus-based oncolytic therapy enhances the secretion of TNF-α and interferon-gamma at the tumor site, inducing the activation and recruitment of neutrophils, eosinophils, and lymphocytes into tumor tissues, thereby potentiating the immune response to eliminate tumor cells [39]. Other studies have shown that following infection with vesicular stomatitis virus (VSV), the number of neutrophils in the bone marrow of mice decreases markedly within 24 h, accompanied by their rapid release and migration to other tissues. At 3 hours post-infection, the proportion of neutrophils in the peripheral blood increases significantly, and over time, neutrophils gradually transition from a mature (CD101^+^) to an immature (CD101^-^) phenotype. At 24 h post-infection, the proportion of neutrophils in the spleen increases significantly. These splenic neutrophils exhibit enhanced antigen-presenting capacity (indicated by increased expression of MHC-II) and elevated secretion of inflammatory cytokines, such as interleukin-20, thereby playing an immune-activating role in oncolytic virotherapy and promoting T cell-mediated antitumor immune responses [40]. The mechanisms of neutrophil-enhanced tumor killing enhancement were illustrated in Figure 3.

#### Activation of immune response

In a study on the treatment of pancreatic ductal adenocarcinoma using VSV, researchers found that VSV-S, constructed by inserting a Smac expression cassette into the VSV genome, could induce remodeling of the TME. This remodeling was primarily characterized by a marked increase in neutrophil infiltration, alongside significant reductions in myeloid-derived suppressor cells (MDSCs) and macrophages. Furthermore, the levels of immunosuppressive factors such as arginase I, TGF-β, and interleukin-10 were substantially decreased. Collectively, these changes shifted the TME from an immunosuppressive to an immunostimulatory state, thereby enhancing the antitumor immune response [41]. Another study demonstrated that a novel oncolytic adenovirus (ADV) could trigger effector mechanisms of immunoglobulin G1 (IgG1) and immunoglobulin A1 (IgA1) by secreting a cross-hybridizing Fc fusion peptide targeting programmed death-ligand 1. This approach activated neutrophils and integrated multiple effector mechanisms, significantly improving tumor cell killing efficiency [42]. In the Raji (Burkitt lymphoma) model, neutrophil depletion markedly diminished the therapeutic efficacy of oncolytic measles virus (MV) [43]. Moreover, neutrophils were found to further enhance the antitumor effects induced by MV through GM-CSF [44]. MV-s-NAP, a recombinant virus constructed using MV and *Helicobacter pylori* neutrophil-activating protein (NAP), exhibited antitumor activity in both subcutaneous and pleural effusion mouse models of metastatic breast cancer. The primary mechanism involves NAP recruiting innate immune cells, including neutrophils and macrophages, to the infection site, promoting the production of reactive oxygen species (ROS) and pro-inflammatory mediators, and thereby inducing a Th1-polarized immune response [45]. The novel oncolytic ADV Adf35 (Uppsala University) expresses NAP and reverses the immunosuppressive TME [46]. Effector memory/effector CD8^+^ T cells (TEM/TE) are key antitumor effectors with strong cytolytic activity and high interferon-gamma secretion [47], but ADV therapy often reduces their TME infiltration. To address this, Gu constructed ADV^NE^, a recombinant oncolytic ADV expressing neutrophil elastase (NE), a serine protease with antitumor activity [48]. ADV^NE^ induces pyroptosis in colorectal cancer cells, releasing HMGB1, which binds to macrophage TLR4 and activates the MyD88-NFκB-NLRP3 pathway, promoting M1 polarization of tumor-associated macrophages [49, 50]. M1 macrophages then enhance TEM/TE infiltration, overcoming the limitation of ADV monotherapy and improving anti-colorectal cancer efficacy [51]. Furthermore, neutrophils within the TME can differentiate into antigen-presenting cells, presenting virus-released tumor antigens to T cells and thereby amplifying the antitumor immune response [52]. Additional studies have demonstrated that modified vaccinia virus induces neutrophil migration primarily via the chemokine receptors CCR1 and CXCR2, promoting neutrophil infiltration and inflammatory responses, and enhancing the vaccinia virus-elicited adaptive immunity [53]. In another study, researchers administered the low-dose oncolytic poxvirus JX-594 via intratumoral injection to patients with advanced metastatic melanoma, then the release of GM-CSF led to a marked elevation in peripheral blood neutrophil levels in a subset of patients, and induced local lymphocyte infiltration and tumor necrosis at the injection site [54]. Table 1 is a summary of the above examples.

**TABLE 1:** Mechanisms of immune response activation by neutrophils.

Oncolytic virus	Mechanisms of neutrophil-mediated anti-tumor effects	References
VSV	Increasing tumor infiltration of neutrophils TME shifted toward immune activation	[41]
Ad-cab	Enhances tumor cell killing by activating neutrophils through IgG1/IgA1 engagement	[42]
MV	Raji model: neutrophils releasing TRAIL and multiple antitumor cytokines to induce tumor cell apoptosis	[43]
	Breast cancer: Recruits and activates neutrophils and other immune cells, induces Th1 polarization and pro-inflammatory cytokine release	[45]
Adf35	Recruits and activates neutrophils and other immune cells to reverse the immunosuppressive tumor microenvironment	[46]
ADV^NE^	Differentiate into antigen-presenting cells within the tumor microenvironment to present tumor antigens to T cells	[47–52]
MVA	CCR1 and CXCR2 induce neutrophil migration and enhance adaptive immunity	[53]
JX-594	Elevated neutrophil levels recruit immune cells	[54]

### Negative regulation mechanism

#### Antiviral activity

As the first line of defense in the immune system [55], neutrophils secrete various antimicrobial and antiviral substances, including myeloperoxidase, defensins, and antimicrobial peptides. Additionally, neutrophils can clear viruses through direct phagocytosis, interaction with other immune cells, or release of cytokines and chemokines [56]. For instance, most oncolytic vaccinia viruses are phagocytosed and degraded by neutrophils in the bloodstream. Depletion of neutrophils using anti-Ly6G antibodies leads to increased accumulation of circulating oncolytic vaccinia viruses in peripheral blood and enhanced viral deposition at tumor sites, thereby potentiating antitumor efficacy [57]. Other studies have shown that following initial administration of VSV, the virus binds to endothelial cells, triggering tumor infection and activating a pro-inflammatory response; however, this initial OV dose also induces neutrophil migration to the tumor and limits viral replication [58]. Persistently elevated neutrophil levels induced by OVs may also cause systemic inflammation, which can inhibit both OV replication and adaptive immune responses, thereby attenuating the antitumor effects of oncolytic virotherapy [39].

**FIGURE 4:**
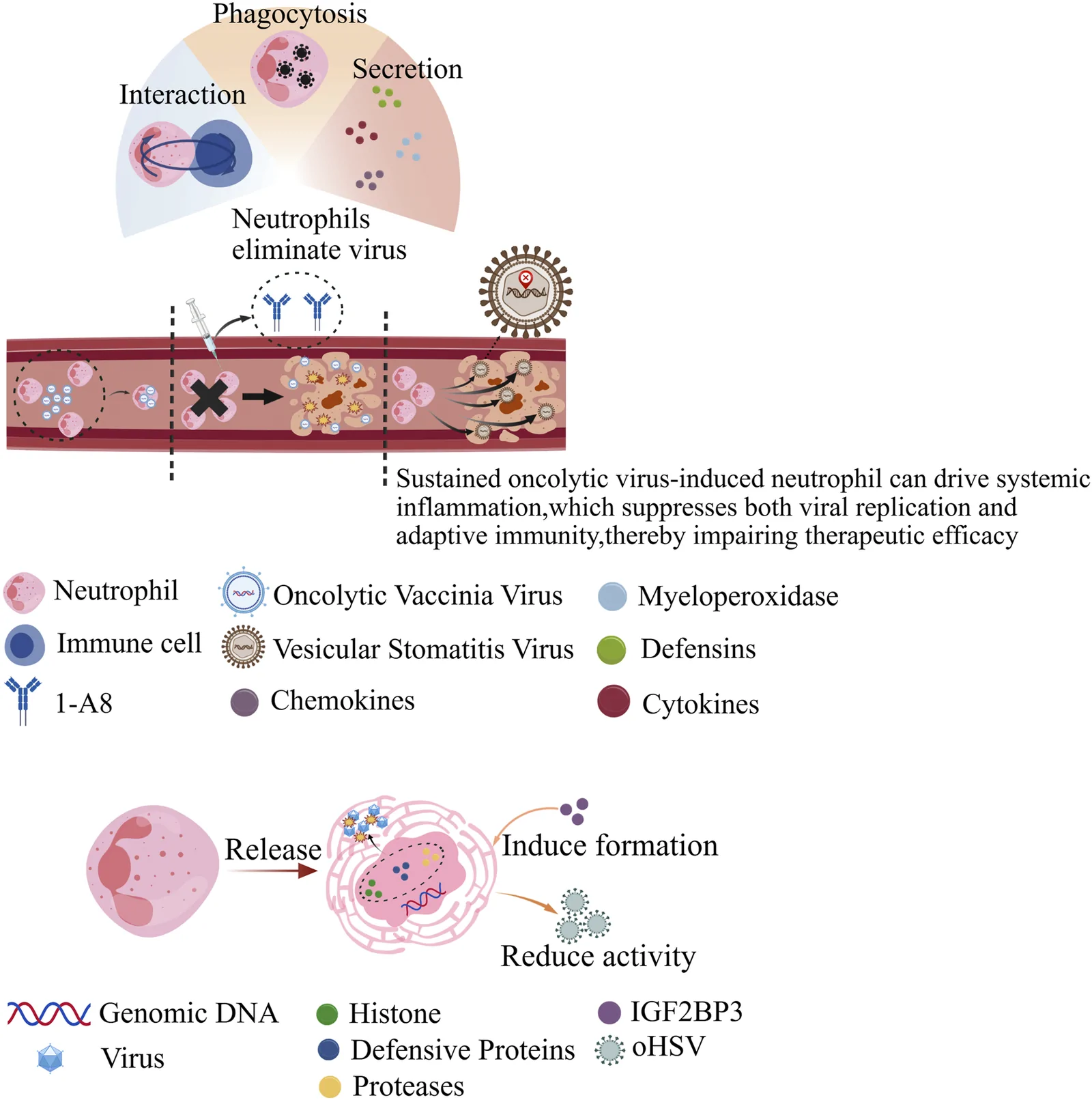
Mechanisms of neutrophil antiviral activity. Created with BioGDP.com [8].

Neutrophils can also suppress the activity of OVs through the release of neutrophil extracellular traps (NET) [59], while simultaneously promoting tumor cell proliferation and invasion. The research team led by Minfeng Shu at Fudan University demonstrated that in oHSV therapy, upregulation of insulin-like growth factor 2 mRNA-binding protein induces NET formation, thereby reducing the oncolytic activity of oHSV [60]. NETosis is a form of inflammatory cell death in neutrophils, characterized by the release of extracellular structures composed of genomic DNA, histones, defense proteins, and proteases [61]. These structures not only trap viruses but also contribute to viral inactivation through their associated granular proteins [62]. The mechanisms of neutrophil antiviral activity were illustrated in Figure 4.

#### Mediating immunosuppressive microenvironment

**FIGURE 5:**
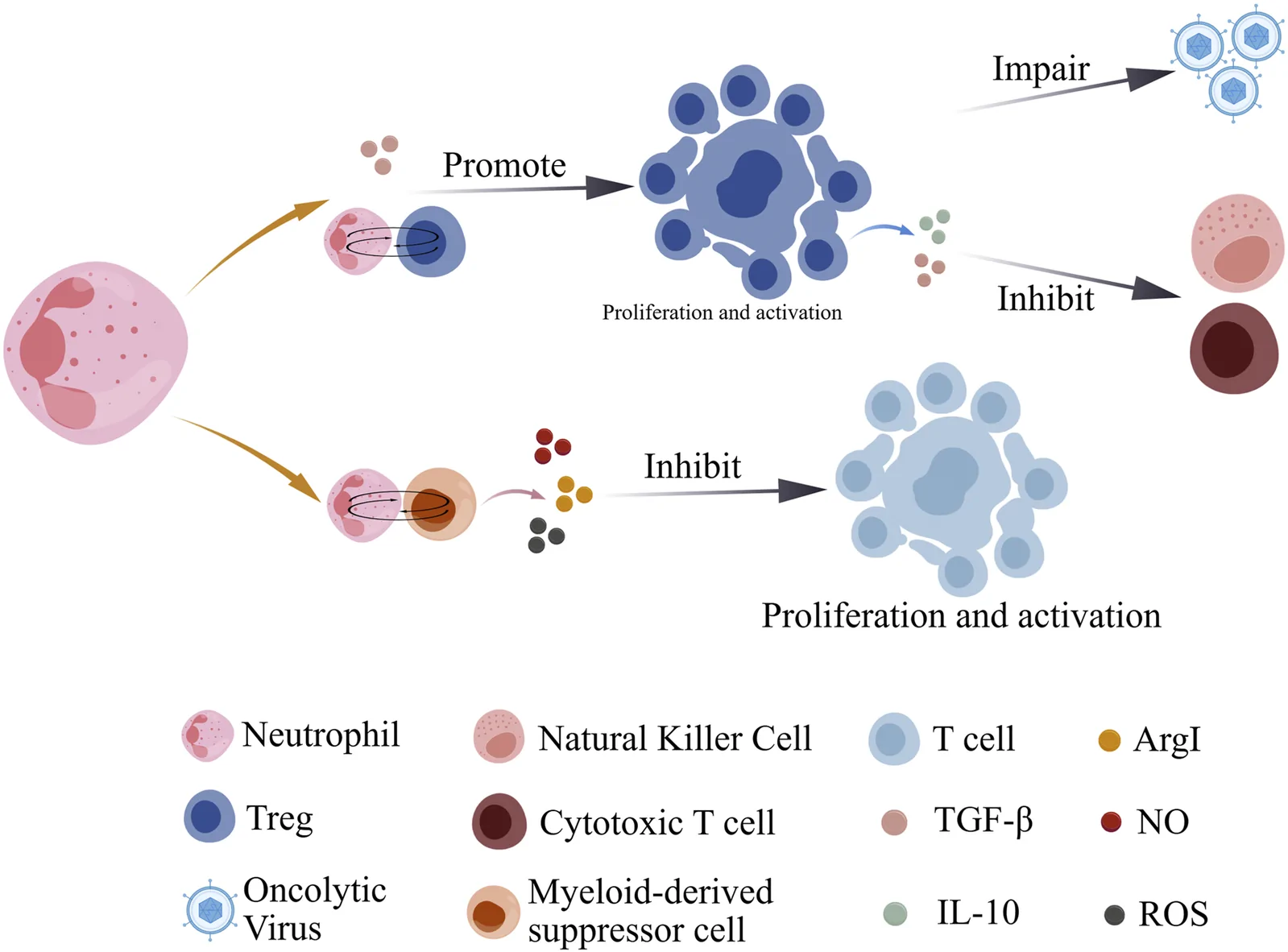
Mechanisms of neutrophil-mediated immunosuppressive microenvironment. Created with BioGDP.com [8].

Neutrophils in the TME induce the expansion of regulatory T cells (Tregs), thereby inhibiting the anti-tumor immune response [63] and attenuating the efficacy of OVs. Tregs are a subset of cells with immunosuppressive functions that suppress the activity of cytotoxic T cells and natural killer cells by secreting inhibitory cytokines such as interleukin-10 and TGF-β. Neutrophils also promote the proliferation and activation of Tregs through the secretion of cytokines or direct interaction with Tregs [14]. Furthermore, neutrophils can interact with MDSCs, facilitating their maturation and functional competence. MDSCs [64] accumulate extensively in the TME [65] and subsequently inhibit T cell proliferation and activation by secreting substances such as arginase I, nitric oxide, and reactive oxygen species [66], which represents one of the core mechanisms by which they mediate tumor immune escape. The mechanisms of neutrophil-mediated immunosuppressive microenvironment were illustrated in Figure 5.

## Discussion

Neutrophils serve as the first line of defense in the immune system. The recently proposed consensus roadmap for neutrophil classification indicates that these cells are not a homogeneous population, but can depart from their intrinsic developmental trajectories and undergo deterministic reprogramming within specific local microenvironments. During the TME remodeling induced by OV therapy, tumor-infiltrating neutrophils not only shift from their original antiviral state but can also be polarized into antitumor effector cells capable of directly killing tumor cells or highly expressing MHC-II molecules and co-stimulatory markers. The mechanistic role of neutrophils in OV therapy is dual-edged. On one hand, neutrophils can ferry OVs across physiological barriers and activate anti-tumor immune responses. On the other hand, they can inactivate OVs through the release of NETs and collaborate with Tregs and MDSCs to establish an immunosuppressive network that undermines OV therapeutic efficacy. A study reported that neutrophil depletion using anti-Ly6G antibodies significantly prolongs the half-life of OVs in the peripheral blood and substantially enhances their targeted delivery to tumor sites [57]. However, depleting neutrophils or artificially blocking their recruitment to sites of pathology would clinically recapitulate the fatal condition of leukocyte adhesion deficiency, then lead to a complete breakdown of host defense against bacterial and fungal pathogens and predispos patients to acute, uncontrollable, and life-threatening infections. Therefore, the future optimization strategy should not be the blunt depletion of the entire neutrophil compartment, but rather a shift toward more refined and precise immunomodulatory approaches. For example, targeting specific chemokine receptors such as CXCR2 or selectively interfering with particular immunosuppressive subsets can block their recruitment while preserving normal immune surveillance in the periphery. Multiple preclinical and clinical studies are progressively validating the feasibility of this strategy. In a phase II clinical trial, the CXCR2 antagonist navarixin in combination with pembrolizumab for the treatment of advanced solid tumors induced neutropenia without associated fever or infection [67]. Although the combination regimen was prematurely terminated due to insufficient efficacy, the favorable safety profile and neutrophil-modulating capacity of navarixin provide an important foundation for future combination with OV therapy. Similarly, the selective PAD4 inhibitor JBI-589 transcriptionally downregulates CXCR2 expression, thereby precisely blocking neutrophil migration into the TME, suppressing tumor progression, and enhancing the efficacy of immune checkpoint inhibitors [68]. Another CXCR2 antagonist, SB225002, blocks radiotherapy-induced activation of the CXCR2-CXCL signaling axis, inhibits neutrophil recruitment, and reduces TGF-β expression, thereby reversing the polarization of neutrophils toward a pro-tumor N2 phenotype and alleviating the immunosuppressive microenvironment [69]. These drugs that precisely control neutrophil migration and polarization offer powerful tools for combining with OV therapy. This conceptual shift from “global depletion” to “precision modulation” may represent the key to advancing OV-based combination immunotherapy toward safe and effective clinical application. Moreover, the complexity and heterogeneity of the TME can significantly influence the efficacy of OV therapy. Neutrophil responses vary from patient to patient, and such individual differences make it very difficult to predict treatment outcomes. Therefore, personalized treatment strategies are needed.

Future translational research should focus on potential regulatory directions, including temporally dynamic regulation of neutrophil functions, spatial blockade of their pathological recruitment to tumor sites, synergistic combination with immune checkpoint inhibitors, and clinically precise patient stratification based on neutrophil-related biomarkers. A thorough mechanistic understanding of neutrophil behavior in the context of OV therapy is importance for optimizing this therapeutic modality and advancing its clinical applicability.
