# Supplementary material for: The role and mechanism of neutrophils in oncolytic virus therapy
Source: Exp Biol Med (Maywood). 2026 Aug 6;251:11192. doi: 10.3389/ebm.2026.11192 (PMC13489981; doi:10.3389/ebm.2026.11192)
Supplement: Supplementary file 1 [file Table1.docx]

Supplementary table 1. Oncolytic viruses in clinical trials

| Virus type | Oncolytic virus name | Conditions | Phase | Clinical Trial Number | Status |
| --- | --- | --- | --- | --- | --- |
| HSV | rHSV2hGM-CSF | Melanoma Stage IV | Phase1 | NCT05068453 | Completed |
| HSV | Talimogene laherparepvec | Triple Negative Breast Cancer | Phase 1/2 | NCT02779855 | Completed |
| HSV | G207 | recurrent or progressive brain tumor | Phase1 | NCT02457845 | Completed |
| HSV | G207 | Recurrent or Refractory Cerebellar Brain Tumors | Phase1 | NCT03911388 | Recruiting |
| HSV | G207 | Malignant Glioma | Phase1 | NCT00157703 | Completed |
| HSV | HF10 | Pancreatic Cancer Stage III/Ⅳ | Phase1 | NCT03252808 | Active，not recruiting |
| HSV | OH2 | Melanoma | Phase3 | NCT05868707 | recruiting |
| HSV | JL15003 | Recurrent Glioblastoma Multiforme | Phase1/2 | NCT07501559 | recruiting |
| HSV | NV1020 | Colorectal Cancer、Liver Neoplasms | Phase1/2 | NCT00149396 | Completed |
| oncolytic adenovirus | Enadenotucirev | Locally Advanced Rectal Cancer | Phase1 | NCT03916510 | Completed |
| oncolytic adenoviru | ADV/HSV-tk | Metastatic Non-small Cell Lung Cancer、Metastatic Triple-negative Breast Cancer | Phase2 | NCT03004183 | Completed |
| oncolytic adenoviru | LOAd703 | Pancreatic Cancer | Phase1/2 | NCT02705196 | Completed |
| oncolytic adenoviru | LOAd703 | Pancreatic Adenocarcinoma、Ovarian Cancer、Biliary Carcinoma、Colorectal Cancer | Phase1/2 | NCT03225989 | Completed |
| oncolytic adenoviru | H101 | Genital Neoplasms, Female | Phase2 | NCT05051696 | Active，not recruiting |
| oncolytic adenoviru | ICOVIR5 | Solid Tumors | Phase1/2 | NCT01844661 | Completed |
| oncolytic adenoviru | AloCELYVIR | Diffuse Intrinsic Pontine Glioma、  Medulloblastoma, Childhood, Recurrent | Phase1/2 | NCT04758533 | Active，not recruiting |
| oncolytic adenoviru | NSC-CRAd-Survivin-pk7 | Glioma | Phase1 | NCT03072134 | Completed |
| oncolytic adenoviru | Oncolytic Adenovirus Ad5-DNX-2401 | Glioma | Phase1 | NCT03896568 | Recruiting |
| oncolytic adenoviru | Oncolytic Adenovirus Ad5-DNX-2401 | Brainstem Glioma | Phase1 | NCT03178032 | Completed |
| oncolytic adenoviru | CAdVEC | Bladder Cancer、Head and Neck Squamous Cell Carcinoma、Cancer of the Salivary Gland、Lung Cancer、Breast Cancer、Gastric Cancer、Esophageal Cancer、Colorectal Cancer、Pancreatic Adenocarcinoma、Solid Tumor | Phase1 | NCT03740256 | Recruiting |
| vaccinia virus | GL-ONC1 | Cancer of Head and Neck | Phase1 | NCT01584284 | Completed |
| vaccinia virus | GL-ONC1 | Ovarian Cancer、Peritoneal Carcinomatosis、Fallopian Tube Cancer | Phase1/2 | NCT02759588 | Completed |
| vaccinia virus | GL-ONC1 | Ovarian Cancer、Peritoneal Carcinomatosis、Fallopian Tube Cancer | Phase3 | NCT05281471 | Recruiting |
| vaccinia virus | Pexa Vec | Hepatocellular Carcinoma | Phase3 | NCT02562755 | Completed |
| vaccinia virus | KM1 | Ovarian Cancer | Phase1 | NCT05684731 | Recruiting |
| vaccinia virus | JX-594 | Melanoma、Lung Cancer、Renal Cell Carcinoma、Squamous Cell Carcinoma of the Head and Neck | Phase1 | NCT00625456 | Completed |
| measles virus | MV-NIS | Ovarian Cancer、Peritoneal Carcinomatosis、Fallopian Tube Cancer | Phase1/2 | NCT02068794 | Active，not recruiting |
