# Supplementary material for: The role and mechanism of neutrophils in oncolytic virus therapy
Source: Exp Biol Med (Maywood). 2026 Aug 6;251:11192. doi: 10.3389/ebm.2026.11192 (PMC13489981; doi:10.3389/ebm.2026.11192)
Supplement: Supplementary file 2 [file Table2.docx]

Supplementary table 2. Neutrophil subsets in tumor therapy

| Subset Name | Impact on Tumor Therapy | Referenc-es |
| --- | --- | --- |
| Low-Density Neutrophils (LDNs / PMN-MDSCs) | Significantly accumulated in peripheral blood of patients with metastatic breast cancer compared to healthy donors or non-metastatic breast cancer patients. Elevated levels correlate with rapid disease progression, shortened survival, and therapy resistance. | [18] |
| CD16ʰⁱ / CD62Lˡᵒ Immunosuppressive Neutrophils | Not yet clarified |  |
| Antigen-Presenting Neutrophils | CD74ʰⁱᵍʰSiglecFˡᵒʷ subset (mouse) and CD74ʰⁱᵍʰCD63ˡᵒʷ neutrophils (human) highly express CD74, MHC-I/II, and CD86, and inhibit non-small cell lung cancer (NSCLC) progression. | [19] |
| Aged Neutrophils (Senescent-like Neutrophils) | Aged neutrophils (RLSNs, Retnlg⁺Lcn2⁺ senescence-like neutrophils) promote tumor growth in male bladder cancer by mediating immunosuppression. | [20] |
| Reverse Transendothelial Migration Neutrophils (rTEM Neutrophils) | Not yet clarified |  |
| Single-Cell Transcriptome-Defined Subsets (G0 ~ G5) | In prostate cancer, the Neu_2 subset highly expresses immunosuppressive cytokines, correlating with shorter biochemical recurrence time, higher infiltration of Tregs and M2-TAMs. | [21] |
